# Curcumin, A Potential Therapeutic Candidate for Anterior Segment Eye Diseases: A Review

**DOI:** 10.3389/fphar.2017.00066

**Published:** 2017-02-14

**Authors:** Xiu-Fen Liu, Ji-Long Hao, Tian Xie, Nour Jama Mukhtar, Wiley Zhang, Tayyab Hamid Malik, Cheng-Wei Lu, Dan-Dan Zhou

**Affiliations:** ^1^Department of Ophthalmology, The First Hospital of Jilin UniversityChangchun, China; ^2^Department of Neurosurgery, The People’s Hospital of Jilin ProvinceChangchun, China; ^3^Department of Molecular Pathology, Icahn School of Medicine at Mount Sinai, ManhattanNY, USA; ^4^Department of Gastroenterology, The First Hospital of Jilin UniversityChangchun, China; ^5^Department of Radiology, The First Hospital of Jilin UniversityChangchun, China

**Keywords:** curcumin, corneal diseases, dry eye, conjunctivitis, anterior uveitis, pterygium, cataract, glaucoma

## Abstract

Curcumin, the major curcuminoid of the turmeric, has been extensively used in many countries since ancient time for preventing and/or treating a multitude of diseases. This review is to illustrate the researches on the properties of curcumin and its potential therapeutic efficacy in major anterior segment eye diseases. The bio-medical potential of curcumin is restricted because of its low solubility and digestive bioavailability. This review will discuss promising research in improving curcumin bioavailability through structural modification. *In vitro* and *in vivo* research made progress in studying the beneficial effects of curcumin on major anterior segment eye diseases, including anti-angiogenesis effect in corneal diseases; anti-inflammation or anti-allergy effects in dry eye disease, conjunctivitis, anterior uveitis; anti-proliferation and pro-apoptosis effects in pterygium; anti-oxidative stress, anti-osmotic stress, anti-lipid peroxidation, pro-apoptosis, regulating calcium homeostasis, sequestrating free radicals, protein modification and degradation effects in cataracts; neuroprotective effects in glaucoma. Curcumin exhibited to be a potent therapeutic candidate for treating those anterior segment eye diseases.

## Introduction

Curcumin is a yellow-colored polyphenol that is isolated from the plant *Curcuma-Longa* and is the principal curcuminoid of the popular spice turmeric. It has been used in various curcumin-based products including energy drinks, ointments, capsules, tablets, soaps, cosmetics, and traditional medicines for centuries. Curcumin is extensively used as a supplement in China, India, USA, South Africa, Pakistan, Japan, Thailand, Korea, Siddha, and Chinese medicine for the management of various diseases and conditions, such as wounds, inflammation, and cancers (Strimpakos and Sharma, 2008; Aggarwal and Sung, 2009; Prasad et al., 2014). Curcumin has been considered as an effective drug for various respiratory conditions in traditional Chinese medicine, including allergy, asthma, bronchial hyperactivity and other disorders (sinusitis, coryza, cough, anorexia, and hepatic diseases) (Rahman et al., 2006). During the last three decades, various studies concluded that curcumin has anti-oxidant, anti-inflammatory, anti-angiogenic, and wound-healing effects (Maheshwari et al., 2006; Zhang et al., 2015).

The pathologic mechanisms of major blinding anterior segment eye diseases, such as corneal NV, glaucoma, and cataracts, are often related to inflammation, oxidative stress-mediated response, and angiogenesis. There is substantial evidence that suggest the potential health benefits of curcumin in diets and drugs to prevent the vision threatening eye diseases (Huynh et al., 2013). This review summarizes the mechanisms underlying the effects of curcumin on anterior segment eye diseases.

## Methodology

The PubMed database was searched with the terms “curcumin,” “curcuminoid”, “curcuminoids”, “Curcuma longa,” “turmeric”, “haridra”, “diferuloylmethane”, and “eye”, “ocular”, either alone and in combination. Articles related with anterior segment eye diseases were picked out manually. All articles with English abstract were included, including those published in other language.

## Biological Activities of Curcumin

Curcumin was discovered in 1815, and subsequently identified as 1,7-bis- (4-hydroxy-3-methoxyphenyl)-1,6- heptadiene-3, 5-dione that exhibits keto-enoltautomerism, having an enol form in alkaline media and a keto form in neutral and acidic solutions (Anand et al., 2008). It is supposed to play an important role in many animal disease models. This polyphenol has been considered as an efficacious and safe agent in clinical trials, and curcumin has been approved as a “generally regarded as safe” compound by the U.S. Food and Drug Administration.

Anterior segment eye diseases include conjunctivitis associated with inflammation, cataracts associated with hyperglycemia and oxidative stress, pterygiums associated with excessive proliferation, and other diseases. Varieties of functions of curcumin have also been explored recently, including its anti-inflammatory effects, anti-oxidant activity, hypoglycemic effects, and anti-tumor (breast, prostate, lung, pancreas, ovary, bladder, cervix, head and neck, brain, kidney, and skin) effects (Chen et al., 2006; Strimpakos and Sharma, 2008; Prasad et al., 2014; Bimonte et al., 2015; Zhang et al., 2015) Curcumin can also induce cell death in human uveal melanoma cells through mitochondrial pathway (Lu et al., 2010). All of aforementioned bio-functions contribute to the potential beneficial effects of curcumin on anterior segment eye diseases.

## Synthetic Analogs of Curcumin in Ocular

It is still considered a challenge for topical therapy to corneal diseases because tear fluids can wash the eye drops away rapidly (Joseph et al., 2016). At same time, the anatomical structure of the cornea causes a natural barrier for drug to penetrate through the cornea into anterior chamber (Chang et al., 2001). Though its safety and efficacy well-established, so far curcumin has not been applied as a therapeutic drug, perhaps, due to its relatively low aqueous solubility (Anand et al., 2007). However, a lot of reports mention that bioavailability might not be a problem. Different types of formulations that have been made with curcumin are to enhance bioavailability through various strategies. The thermo-sensitive gelling agent and nanogel combining CNLC has been designed to enhance the potentials for ocular permeation capacity of curcumin. The developed Curcumin-CNLC-GEL could significantly augment the bioavailability of curcumin and sustain drug concentration in aqueous humor after dispensation in comparison with that of the control group. Those results indicated that Curcumin-CNLC-GEL could become a potential formulation for the enhancement of solubility of curcumin in the aqueous humor by enhancing corneal diffusion and retaining capacity (Liu et al., 2016). Another study reported that methoxypoly (ethylene glycol)-poly (ε-caprolactone) diblock copolymers (MePEG-PCL) nanoparticle of curcumin enhances retention of curcumin in the cornea, and improves corneal NV over curcumin (Pradhan et al., 2015). Jie et al. tested an ophthalmic in situ gel made of curcumin-loaded nanoparticles (Cur-BSA-NPs-Gel), and found that curcumin-loaded albumin nanoparticles (Cur-BSA-NPs-Gel) scored superior sustained-release result, and concluded albumin nanoparticles (Cur-BSA-NPs-Gel) exhibit little effects on the gel structure *in vitro*. The *in vivo* study also indicated that the formulation might greatly increase bioavailability of the curcumin in the aqueous humor. Therefore, the curcumin-loaded albumin nanoparticles (Cur-BSA-NPs-Gel) system depicted that an ophthalmic delivery system that may prolong drug retention time and enhanced ocular bioavailability (Lou et al., 2014). Ion-sensitive curcumin-loaded Pluronic P123 (P123)/D-a-tocopheryl polyethylene glycolsuccinate (TPGS) mixture of micelle in situ gels (CUR-MM-ISGs) prepared by Yu et al. prolonged ocular residence time and greatly enhanced cornea permeability, and these findings showed that the biocompatible CUR-MM-ISGs had significant potentials for effective ophthalmic drug therapy (Duan et al., 2015). A novel demethylated curcuminoid compositon exhibited superior anti-inflammatory and neuroprotective efficacy compared to curcuma longa extract. Demethylated curcuminoid composition was classified as mildly irritating to the eye based on the primary eye irritation test on rabbits (Krishnaraju et al., 2009). An adjunctive-to-traditional medicine with Norflo tablets (curcumin-phosphatidylcholine complex) was given two times a day in various recurrent anterior uveitis. It is illustrated that Norflo tablets was well endured and could potentially minimize ocular discomfort several weeks later in almost 80% of patients (Allegri et al., 2010). Other curcumin based formulation was also reported that are made to owing more biological activities than curcumin (Shoji et al., 2008; Liu, 2011; Das and Sahoo, 2012; Steigerwalt et al., 2012; Grama et al., 2013).

## Curcumin As A Promising Therapeutic Candidate for Anterior Segment Eye Diseases

### Cornea Diseases

#### Inhibiting Corneal NV

The cornea is the transparent avascular anterior part of the eye. Corneal NV is a condition in which excessive blood vessels grow into the cornea and is the leading cause of blindness. This condition is trigged by corneal hypoxia, inflammation, and/or limbal barrier dysfunction. Currently, the treatments for corneal NV include steroids, nonsteroidal anti-inflammatory eye drops, fine needle diathermy, photodynamic therapy, and anti-VEGF therapy (Chang et al., 2001). Though, these therapies ameliorate corneal NV to some extent, the side effects cannot be ignored: steroids may cause corneal thinning, ocular hypertension, and cataracts (Sarchahi et al., 2008); NSAIDs may lead to corneal ulceration and perforation (Guidera et al., 2001); fine needle diathermy and photodynamic therapy may cause an inflammatory response (Shakiba et al., 2009; Koenig et al., 2012); and anti-VEGF therapy complications include corneal thinning, reduced epithelial healing (Kim et al., 2008), and epithelial erosion (Oh et al., 2009). Therefore, a safe and effective therapy for corneal NV is needed.

**FIGURE 1:**
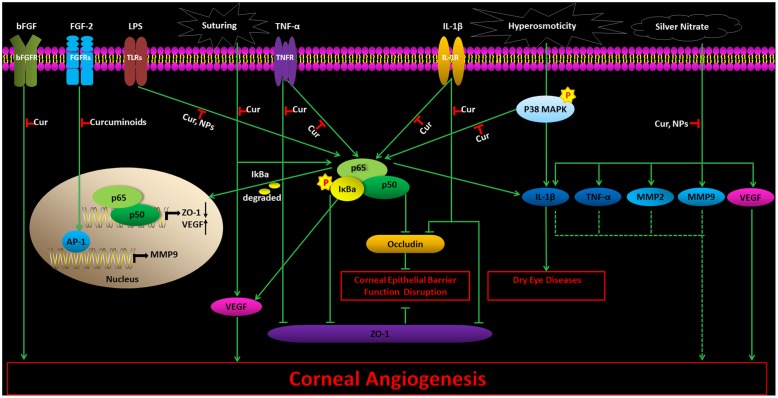
**The effects and mechanisms of curcumin and curcumin NPs on corneal diseases.** Cur, Curcumin; NPs, curcumin nanoparticles. Thin arrows show direct reactions, and dotted arrows indicate indirect reactions.

Vascular endothelial growth factor and basic fibroblast growth factor (bFGF) are supposed to attract and recruit inflammatory cells, leading to corneal NV. Curcumin could inhibit proliferation of primary endothelial cells cultured *in vitro*, either in the presence or absence of bFGF (Arbiser et al., 1998). It was able to prohibit bFGF-induced mouse corneal NV *in vivo* (Arbiser et al., 1998). Suturing-induced rabbit corneal NV could also be suppressed by curcumin, *in vivo* (Kim et al., 2010) via decreasing the VEGF mRNA levels and phosphorylation of NF-κB (Kim et al., 2010). Curcumin NPs might significantly reduce angiogenic sprouting in a dose and time dependent manner in the mouse aortic ring *in vitro*. Curcumin NPs were also able to inhibit NF-κB in LPS-induced corneal cells *in vitro* (Pradhan et al., 2015). It may also prohibit corneal angiogenesis in silver nitrate-induced corneal NV *in vivo* through the mechanism of inhibiting VEGF, inflammatory cytokines [IL-1β, TNF-α], and MMPs (MMP-2 and MMP-9) (Pradhan et al., 2015). Curcuminoids, administered locally or in the diet, could suppress fibroblast growth factor-2 (FGF-2)-induced rabbit corneal NV *in vivo*, by inhibiting DNA binding activity from transcription factor activator protein-1 (AP-1) and gelatinase B promoter activity (Mohan et al., 2000). The effects and mechanisms of curcumin and curcumin NPs on corneal diseases warrant an effective and safe herbal therapy for preventing corneal NV (**Figure 1**).

#### Protecting Corneal Epithelial Barrier

The corneal epithelium provides a strict barrier that is both useful for dependent on tight junctions between adjacent epithelial cells and corneal homeostasis. Barrier function of the corneal epithelium can be undermined by bacterial infection or inflammation, resulting in different conditions of the epithelium and in stromal edema, infection, or melting. Kimura et al. (2008) found that TNF-α or IL-1β could damage the human corneal epithelial cells barrier *in vitro* by affecting the localization of the tight junctions proteins zonula occludens-1 at tight junctions dependent on NF-κB. Curcumin could inhibit both TNF-α and IL-1β induced subcellular localization of occludens-1 through NF-κB inhibition *in vitro*, indicating curcumin may prevent corneal epithelial barrier function disruption related with ocular inflammation (Kimura, 2010).

#### Promoting Corneal Wound Healing

Diabetes increases the risk of corneal diseases, and as high as 50% of diabetic patients suffered from diabetic keratopathy (Zagon et al., 2007), including reduced corneal sensitivity, decreased tear secretion, tear film dysfunction, degeneration of nerve fibers, loss of corneal epithelia, and corneal ulcer (Gao et al., 2015). The current treatments for diabetic keratopathy include artificial tears, antibiotic eye drops, tarsorrhaphy, and bandage contact lens, which helps for corneal wound healing (Abdelkader et al., 2011). However, these measures may not be effective and adequate at accelerating re-epithelialization in diabetes, even if different therapies are used simultaneously. Thus, novel effective methods are required.

Guo et al. reported intranasal nanomicelle curcumin promotes corneal epithelial/nerve wound healing in STZ-induced diabetic mice with corneal epithelium abrasion. The mechanism includes alleviating free radical scavengers, recovering the enhanced accumulation of reactive oxygen species (ROS), decreased mRNA expressions of neurotrophic factors, and increased mRNA expressions of inflammatory cytokines in the cornea. Trigeminal ganglion neurons were also observed in mice with corneal epithelium abrasions. These findings illustrate that intranasal curcumin could effectively promote diabetic corneal epithelial or nerve wound healing. This treatment might be a promising therapy for diabetic keratopathy (Guo et al., 2016).

### Dry Eye Disease

Dry eye disease is one of the most prevalent eye diseases, with an estimated ten million individuals affected in the United States (Niederkorn et al., 2006). Decreased secretion of the tears and rapid tear evaporation are the two contributing factors for dry eye disease. Increased tear osmolality and ocular surface inflammation are involved in the pathogenesis of ocular surface damage during the course of the disease. Different types of anti-inflammatory drugs like topical corticosteroid usually improve the symptoms of the disease in short term trials (Yang et al., 2006). Unfortunately, in the long run, some serious potential side effects limit its use (Lemp, 2007). So far, all the available drugs have some limitations, which call for the need to develop more potent drugs for the treatment of dry eye disease. The possible mechanisms of curcumin on dry eye disease are still unclear. Increased tear osmolality is an important feature of the disease. In a hyperosmotic environment, pro-inflammatory cytokines such as interleukin-6 (IL-6), interleukin-8 (IL-8), and IL-1β are detected in corneal cell lines or dry eye patients (Solomon et al., 2001; Li, 2006; Chotikavanich et al., 2009). Curcumin could inhibit ovalbumin-induced pro inflammatory cytokines [interleukin-4 (IL-4), interleukin-5 (IL-5)] expression in conjunctiva in mice (Chung et al., 2012). Our previous study showed that curcumin guarded against hyperosmoticity-induced IL-1β upregulation in corneal epithelial cell *in vitro* through p38 mitogen-activated protein kinase (MAPK)/NF-κB pathways (Chen et al., 2010), which indicates that curcumin might serve as a promising candidate for treating dry eye disease.

### Conjunctivitis

Conjunctivitis is one of the most frequently occurring ocular diseases worldwide. It can be caused by bacteria, viruses, and allergies. Conjunctivitis is becoming increasingly prevalent due to the resilient bacterial strain infections, changing climate, increased pollen loads, pollution, and the resulting heightened immunological response to the environmental changes. There are many ocular medications for treating conjunctivitis but they are expensive and the long treatment period increases the risk of microbial resistance. An inexpensive and low side-effect treatment is needed, especially in developing countries.

Ophthacare, obtained from the Himalaya Drug Company, is the brand name of a mixed herbal eye drop with 8 different herbs, – including curcuma-longa (rhizome) 1.30% w/v – was reported to have positive effects to treat conjunctivitis, conjunctival xerosis (dry eye), etc. (Biswas et al., 2001). In a previous study, it was reported to have effects in different ophthalmic diseases namely, conjunctivitis, conjunctival xerosis (dry eye), etc. (Biswas et al., 2001). The study showed that OphthaCare can be safely administered in various infective and inflammatory conjunctival diseases, but has still yet to be assessed in a double-blind clinical trial. Haridra (*Curcuma-Longa*) is available freely in India and in tropics, and Haridra Eye Drops was reported to have an important role in treating bacterial conjunctivitis in a clinical study. Bacteriological study shows the Haridra has an active role against Escherichia coli (*E. coli*), St. Aureus, Klebshella, and pseudomonas organisms (Srinivas and Prabhakaran, 1989). Curcumin was reported to suppress ovalbumin-induced allergic conjunctivitis in an experimental mice model (Chung et al., 2012). Mice challenged with ovalbumin via the conjunctival sac following systemic sensitization in aluminum hydroxide had severe allergic conjunctivitis. Pre-administration of curcumin 1h before ovalbumin challenge could markedly inhibit the activation of inducible nitric oxide synthase (iNOS) production in the mice conjunctiva and suppress immunoglobulin E (IgE)-mediated and eosinophil-dependent conjunctival inflammation. Inhibition of IL-4 and IL-5 expression in conjunctiva, cervical lymph nodes, and spleen was observed in mice treated with curcumin, when compared to mice challenged with ovalbumin. These results indicate that curcumin suppresses allergic conjunctivitis through its anti-allergic and anti-inflammation properties.

### Pterygium

Pterygium is an inflammatory and degenerative ocular surface disease in which the conjunctiva on the cornea grows to form fibrous tissue in a triangular shape. The pathogenesis is not completely understood, but recent evidence suggests that pterygium is histologically composed of proliferating fibro vascular tissue and is correlated strongly with exposure to ultraviolet radiation (Yam and Kwok, 2014; Shah et al., 2016). Surgical excision is the first-choice treatment for pterygium, but the high recurrence rate is a burden for patients. Therefore, the treatment of pterygium remains quite controversial. The identification of effective drugs for the treatment of pterygium is urgently needed. Curcumin (20–80 μmol/L) was found to hinder the expression of spreading cell nuclear antigen and to stop the proliferation and cause the cell death of human pterygium fibroblasts both in a dose-and time-dependent manner, indicating that curcumin had a potential therapeutic outcome against pterygium (Zhang et al., 2007). However, the study was restricted to *in vitro* experiment. Biswas et al. (2001) documented that OphthaCare showed an excellent response in the treatment of pterygium.

### Anterior Uveitis

Anterior uveitis is associated with inflammation of the uveal tract (especially the iris) and without intervention can cause blurry vision and permanent damage to the eye. It is the fourth most common cause of blindness in developed countries (Chang and Wakefield, 2002; Merida et al., 2015). The underlying mechanism of uveitis is unclear due to its heterogeneity. Corticosteroid and NSAIDs are the main available treatment for uveitis. However, side-effects (i.e., cataract, secondary glaucoma, anterior, and posterior synechiae) are commonly observed with steroid therapy. Corticosteroid can only provide some short-term relief, but not significant long-term relief. An alternative therapeutic that could relieve inflammation like a corticosteroid, but without any adverse side effects is needed.

It was reported that curcumin could treat chronic anterior uveitis without any adverse side effects (Lal et al., 1999). Curcumin was filled in capsules (375 mg curcumin/capsule) and taken orally by patients with chronic anterior uveitis. Patients took one capsule t.i.d. along with local cyclopegics (e.g., atropine/cyclopentolate1%) improved their vision significantly with decreased aqueous flareand keratic precipitates (Lal et al., 1999).

Meriva (Indena, Milano, Italy) is a product-curcumin formulated with phosphatidylcholine-which improves bioavailability at least 10 times compared to standard curcumin (Marczylo et al., 2007). It exhibited a beneficial effect in the adjunctive therapy of recurrent anterior uveitis with different etiologies, including herpetic (cytomegalovirus, Epstein–Barr virus, varicella zoster virus, and herpes simplex virus) uveitis, autoimmune (overlap syndrome, sarcoidosis, rheumatoid arthritis, and systemic lupus erythematosus) inflammatory ocular disease, and different origin anterior uveitis (seven toxoplasmic etiology, eight unknown origin, four Lyme disease, and three tuberculosis), with the most sensitive being for autoimmune uveitis, and more relapsing against herpetic uveitis (Allegri et al., 2010). Various mechanisms for these beneficial effects of curcumin have been proposed. Topically applied standardized aqueous extract of *Curcuma-Longa* suppresses *E. coli* lipopolysaccharide-induced anterior uveitis in rats (Agarwal et al., 2013) and rabbits (Gupta et al., 2008) by reducing TNF-α activity (Agarwal et al., 2013). Curcumin may also stabilize the lysosomal membranes (Srivastava and Srimal, 1985; Dikshit et al., 1995), inhibit leukotrienes (Flynn et al., 1986; Ammon et al., 1992) and thromboxane B4 (Srivastava and Srimal, 1985). It possesses strong free radical scavenging (Zhao et al., 1989) and anti-oxidant properties (Rajakumar and Rao, 1994). It was found to prompt nitric oxide synthesis in activated macrophages and to inhibit neutrophil activity (Brouet and Ohshima, 1995). Its beneficial effect may be related to the anti-fibrinolytic activity for breaking the anterior and the posterior synechiae (Lal et al., 1999).

Compared with corticosteroids, curcumin exhibits an advantage in lacking adverse side effects in the treatment of chronic anterior uveitis.

### Cataract

Cataracts accounts for more than one third of blindness globally (Resnikoff et al., 2008). Twenty-five percent of people over the age of 65 and 50% of people over the age of 80 have a serious loss of vision due to cataracts (Foster and Resnikoff, 2005). Cataract extraction surgery is the mainstream treatment for cataract. While cataract surgery is considered to be safe and mature, irreversible blindness is a potential risk. There is no recognized medication which can cure or reverse cataract. If cataract onset is delayed by 10 years, it is estimated to reduce the possibility for cataract surgery by 50% (Kupfer, 1985). Thus, much emphasis is being laid on identifying compounds with high effectiveness and low toxicity that can either prevent the onset or delay cataract progression.

#### Anti-oxidative Stress

It is believed that oxidative damage to the eye lens contributes to the development of different kinds of cataracts (Suryanarayana et al., 2005; Manikandan et al., 2010a,b). The primary mechanism for the anti-cataract effect of curcumin is through its antioxidant properties (Manikandan et al., 2009, 2010a, 2011; Radha et al., 2012).

Curcumin may inhibit peroxiredoxin 6 (a pleiotropic oxidative stress-response protein) in cultured human lens epithelial cells (hLECs) *in vitro* (Chhunchha et al., 2011). Bioactive derivatives of curcumin (salicylidenecurcumin CD1 and benzalidenecurcumin CD2) were reported to inhibit the selenite induced cataract by reversing the activity of antioxidant enzymes, decreasing ROS, and increasing the activities of superoxide dismutase (SOD) (Manikandan et al., 2010a; Radha et al., 2012).

Curcumin was found to have a protective effect against cataract development and/or progression in numerous *in vitro* and *in vivo* cataract models (Awasthi et al., 1996; Pandya et al., 2000; Suryanarayana et al., 2003, 2005; Kumar et al., 2005a,b; Raju et al., 2006; Manikandan et al., 2009, 2010a,b, 2011; Grama et al., 2013). It may suppress selenium-induced oxidative stress in rat organ cultured lens and delay the formation of cataracts by inhibiting the non-enzymic antioxidants depletion (Manikandan et al., 2009). Vitamin C is a potent non-enzymic antioxidant, and the level of Vitamin C is high in human lens, suggesting that vitamin C may have a preventive role in cataract progression. The decreased vitamin C levels observed in selenite-induced rat cataracts suggests that weakened non-enzymatic antioxidant defenses may play a role in selenite-induced rat cataracts. Administration of curcumin was found to increase vitamin C levels (Murugan and Pari, 2006). Pretreatment of curcumin may prevent oxidative damage and delay the development of cataracts by increasing superoxidase dismutase and catalase enzyme activity in Wistar rats (Padmaja and Raju, 2004). Curcumin may prevent alterations of protein carbonyls, antioxidant enzymes glutathione peroxidase (GPx), glucose-6-phosphate dehydrogenase (G6PD) and significantly decreased GSH levels, demonstrating curcumin delay the progression of diabetic cataract by preventing hyperglycemia-mediated lenticular oxidative stress in rats (Suryanarayana et al., 2005).

#### Anti-lipid Peroxidation Stress

Lipoperoxides is enhanced in selenite-induced rat cataract *in vivo* (Manikandan et al., 2010b). Hiobarbituric acid-reacting substances (TBARS), commonly measured as a marker of LPO, was also elevated in rat of selenite-induced and STZ-induced cataract *in vivo* (Suryanarayana et al., 2005; Manikandan et al., 2010b), indicating enhanced lipid peroxidation in cataracts. Curcumin significantly delayed the progression and maturation of cataracts in a dose-dependent manner by decreasing LPO and TBARS (Suryanarayana et al., 2005; Manikandan et al., 2010b). GST is a group of multifunctional proteins that performs tasks ranging from catalyzing the detoxification of electrophilic compounds to protecting against peroxidative damage (Sener et al., 1979). GST is decreased in selenite-induced rat cataracts *in vivo*, and curcumin increased the activity of GST to near normal level (Manikandan et al., 2010b). 4-hydroxy-2-transnonenal (4-HNE) is a highly electrophilic product of lipid peroxidation. It was reported that curcumin has a protective effect on organ-cultured lens in 4-HNE induced cataract formation. Curcumin treatment caused an induction of the GST isozymer GST8-8 in rat lenticular epithelium. GST8-8 uses 4-HNE as a favorable substrate, suggesting the beneficial effect of curcumin may be regulated by producing this GST isozyme (Suryanarayana et al., 2005; Manikandan et al., 2010b). GSH is believed to prevent the cells from lipid peroxidation. GSH is known as a free radical scavenger, a cofactor for many enzymes and a co-substrate for glutathione peroxidase (GPx) activity (Gregus et al., 1996). Selenite increases lipid peroxidation in rat lens, leading to downregulation of GSH in the lens. Curcumin pretreatment resulted in maintaining normal GSH levels that was detected in tissues and serums in selenite treated lenses *in vivo*, indicating the protective role of curcumin against oxidative stress (Manikandan et al., 2010b).

Vitamin E, a natural lipid soluble antioxidant, can maintain the integrity of cell membrane and vital membrane functions by inhibiting lipid peroxidation. Vitamin E was reported to have a protective effect in delaying galactose-induced cataract formation and aminothiazole-induced cataract formation in rabbits, inhibiting the lipid photo-peroxidation in lens, limiting radiation related lenticular damage, and preventing diabetic cataract or heat-induced cataract formation (Creighton et al., 1985; Gregus et al., 1996). The level of vitamin E in lens homogenate was decreased in rat treated with selenite alone, and early administration of curcumin produced a protective effect on vitamin E levels (Manikandan et al., 2010b). Curcumin in a 0.01% dose, supplemented with vitamin-E, inhibited galactose-induced rat cataract *in vivo* by inhibiting lipid peroxidation (Raju et al., 2006).

#### Anti-osmotic Stress

Aldose reductase, a vital enzyme for polyol pathway, was significantly increased in STZ-induced diabetic rat lens. Curcumin normalized AR activity *in vivo*, indicating curcumin is effective against osmotic stress caused by hyperglycemia (Suryanarayana et al., 2005; Grama et al., 2013).

#### Protein Modification and Degradation

Studies have shown that even in the earliest stages of cataract formation, the pattern of lens proteins changed (Manikandan et al., 2010a). Significant decreases were found in β crystallins, gamma (γ)-crystallins, and a high molecular weight (HMW) aggregate peak in STZ-induced diabetic rat lens by High Performance Liquid Chromatography (HPLC). The proportion of cross-linked and aggregated proteins were detected to be increased in the soluble protein in STZ-induced diabetic rat lens, suggesting either aggregation or cross-linking might or any unknown factor might be causing protein modification and degradation in diabetic cataractous lens, leading to the formation of HMW aggregates. The insolubility of otherwise soluble protein and alterations in protein profile can result in lens opacification (Kumar et al., 2005b; Suryanarayana et al., 2005). Curcumin and turmeric remarkable alleviated those protein changes, indicating that curcumin and turmeric has a protective effect against diabetic cataract in rats (Kumar et al., 2005b; Suryanarayana et al., 2005). Heat shock protein 70 (Hsp 70), αA-crystalline, αB-crystallin were the predominant constituent proteins which maintain eye lens transparency, and were increased in cataracts. Curcumin could suppress the expression of Hsp 70, αA-crystalline, and αB-crystallin in STZ or selenite-induced cataracts in rat. (Kumar et al., 2005a; Manikandan et al., 2011).

#### Calcium Homeostasis

Although the precise mechanisms of cataracts are not fully elucidated, a plausible mechanism might be that constant oxidative stress causes progressive deterioration of Ca^2++^ homeostasis (Hightower and McCready, 1989). Ca^2+^ is essential for maintenance of lens transparency, and increased Ca^2+^ could activate calpain which can degrade crystallins in lens, leading to cataract formation (Shearer and David, 1982; Thiagarajan and Manikandan, 2013). It was reported that Ca^2+^ ATPase may participate in regulating Ca^2+^ homeostasis in lens (Manikandan et al., 2010a). Curcumin may decrease selenite-induced increase of Ca^2+^ concentration in selenium-induced rat cataract model *in vivo* by increasing the activities of Ca^2+^ ATPase and thus lowering the Ca^2+^ to an almost normal level (Manikandan et al., 2010a).

#### Anti-nitrosative Stress and Scavenge Free Radicals

Excessive free radical generation (NO, OH^-^, O^2-^) has been identified as one of the major etiological factors of cataracts (Spector, 1995). An excess of NO, produced by inducible nitric oxide synthases (iNOS), is thought to cause cell injury by nitrosative stress and contribute to cataract formation (Ito et al., 2001; Ornek et al., 2003; Manikandan et al., 2010a). OH^-^ is also a highly reactive free radical that contributes to lens crystalline modification (Fu et al., 1998). OH^-^ could react with NO, generating more reactive compounds (Graham et al., 1993). Curcumin has been reported to be able to sequestrate free radicals, and scavenge NO, OH^-^, O^2-^ in isolated rat peritoneal macrophage *in vitro* (Joe and Lokesh, 1994). Curcumin prevented uncontrolled generation of free radicals by inhibiting the production of iNOS in selenium-stimulated organ cultured lens of rat pups *in vitro* (Manikandan et al., 2009). Pretreatment of curcumin was found to prevent free radical generation in selenium-induced cataract in rat pups *in vivo* (Manikandan et al., 2010a).

#### Inhibit Proliferation and Induce Apoptosis

Lens cell membrane damage is considered as one of the early signs of cataractogenesis, resulting in changes in intraocular metabolism and composition (Vrensen, 1995). Suppressing the proliferation and inducing apoptosis of lens epithelial cells is the primary goal in preventing cataractogenesis. Curcumin could inhibit proliferation of human lens epithelial B3 (HLE-B3) cells cultured *in vitro* (Hu et al., 2012). It may also induce irreversible apoptosis in bovine lens epithelial cells cultured *in vitro*, through attenuating mitochondrial transmembrane potential in cytoplasm and decreasing DNA content in nucleus (Huang et al., 2006). Further *in vivo* experiments are needed to validate curcumin’s effects on proliferation and apoptosis in lens epithelial cells.

Thus, as a viable food-based pharmacologic drug, numerous studies (summarized in **Table 1**) have demonstrated that curcumin may protect again cataracts and may serve as an effective and low toxic medication for the prevention and treatment of cataract.

**Table 1:** Selected observational studies on the relationship between curcumin and cataract models.

Type	Models	Oberservation	Results	Reference
*In vitro*	Cultured human LEC	Effect of curcumin on peroxiredoxin 6 in ROS-induced oxidative stress-response in human LEC	Curcumin protects LEC by upregulating peroxiredoxin 6 transcription via invoking specificity protein 1 (Sp1) activity against proapoptotic stimuli.	Chhunchha et al., 2011
	Cultured bovine LEC	Effect of curcumin on apoptosis of LEC	Curcumin induced apoptosis of LEC by decreasing of DNA content in LEC nucleus and collapsing of DeltaPsim in cytoplasm.	Huang et al., 2006
	Rat organ cultured lens	Effect of curcumin and its derivatives (CD1, CD2) on selenite-induced cataract	Curcumin and its derivatives (CD1, CD2) are beneficial against selenite-induced cataract by reversing the activity of antioxidant enzymes and calcium homeostasis to near normal levels in lens.	Radha et al., 2012
	Rat organ cultured lens	Effect of curcumin on 4-HNE-induced opacification of lens	Lens from diatary curcumin-treated rats were much more resistant to 4-HNE-induced opacification than control group.	Awasthi et al., 1996
	Rat organ cultured lens	Effect of curcumin on selenium-induced oxidative stress in lens	Curcumin suppressed oxidative stress and cataract formation, prevented uncontrolled generation of free radicals, and inhibited iNOS expression.	Manikandan et al., 2009
*In vivo*	Selenium-induced rat cataract	Effect of curcumin on Ca^2+^ ATPase in selenium-induced cataract	Diatary curcumin prevented selenium-induced Ca^2+^ ATPase activation and inhibited cataract.	Manikandan et al., 2010a
	Selenium-induced rat cataract	Effect of curcumin on αA- and αB-crystallin and heat shock protein 70 in selenite-induced cataract	Diatary curcumin decreased selenium-induced the αA- and αB-crystallin and Hsp 70 production.	Manikandan et al., 2011
	Selenium-induced rat cataract	Antioxidant effect of curcumin on selenium-induced cataract	Dietary curcumin prevented oxidative damage and delay the development of cataract by attenuating lipid peroxidation, xanthine oxidase enzyme activity and increasing superoxidase dismutase and catalase enzyme activity.	Padmaja and Raju, 2004
	Selenite-induced rat cataract	Antioxidant effect of curcumin on selenite-induced cataract	Diatary curcumin decreased LPO, enzymic antioxidants, and nonenzymic antioxidants induced by selenite.	Manikandan et al., 2010b
	Naphthalene-induced rat cataract	Effect of curcumin on naphthalene-induced opacification of lens	Dietary curcumin alleviated naphthalene-induced cataract by attenuating apoptotis of LECs.	Pandya et al., 2000
	Galactose-induced rat cataract	Effect of curcumin on galactose-induced cataract	Diatary curcumin delayed the onset and maturation of cataract by antioxidant and antiglycating effects, as it inhibited lipid peroxidation, AGE-fluorescence, and protein aggregation.	Suryanarayana et al., 2003
	Galactose-induced rat cataract	Effect of vitamin-E and curcumin on galactose-induced cataract	Combination of diatary vitamin-E and curcumin delayed the on the onset and maturation of galactose-induced cataract with an antioxidant effect, as it inhibited lipid peroxidation and contributed to a distinct rise in reduced GSH content.	Raju et al., 2006
	STZ-induced rat diabetic cataract	Effect of curcumin and its source (turmeric) on STZ-induced diabetic cataract	Dietary curcumin delayed the progression of cataract, with reversed change in lipid peroxidation, reduced GSH, protein carbonyl content and activities of antioxidant enzymes, preventing aggregation and insolubilization of lens proteins due to hyperglycemia.	Suryanarayana et al., 2005
	STZ-induced rat diabetic cataract	Effect of curcumin NPs on STZ-induced diabetic cataract	Oral nanocurcumin was effective than curcumin in delaying diabetic cataracts in rats, attributed to its ability to intervene protein insolubilization, polyol pathway, protein glycation, crystallin distribution, and oxidative stress.	Grama et al., 2013
	STZ-induced rat diabetic lens	Effect of curcumin on αA- and αB-crystallins in lens	Dietary curcumin attenuated the enhanced expression of αB-crystallin in lens induced by STZ.	Kumar et al., 2005a
	STZ-induced rat diabetic lens	Effect of curcumin on α-crystallin chaperone activity in lens	αH- and αL-crystallins isolated from curcumin fed diabetic rat lens had shown improved chaperone-like activity as compared to control group.	Kumar et al., 2005b


*LEC, lens epithelial cells; STZ, Streptozotocin.*

### Glaucoma

As a chronic, progressive, irreversible optic nerve neuropathy, glaucoma has already affected more than 60 million people worldwide by 2010 (Quigley and Broman, 2006), and the number continues to grow. It is characterized by persistent loss of retinal ganglion cells, thinning of the retinal nerve fiber layer, and progressive loss of the vision field (Quigley, 1993). However, the exact underlying pathological mechanisms remain unclear. Elevated intraocular pressure is the most critical risk for glaucoma, and it was reported to cause damage to the optic nerve through retinal ganglion cell apoptosis (Ritch, 2007). The most important clinical treatment is lowering the intraocular pressure, using eye drops, oral medications or surgeries. But lowering the intraocular pressure only slows down glaucoma progress, rather than preventing it. In some cases, even when eye pressure has been lowered to normal levels, glaucoma progresses anyway. Thus, neuroprotective agents are desired to prevent or limit or even recover the damage to the optic nerve.

Curcumin was reported to possess neuroprotective properties, which may be effective in the prevention and treatment of glaucoma. In a chronic high intraocular pressure *in vivo* rat model, pretreatment of curcumin was correlated with significantly increased cell viability of BV-2 microglia and the increase presence of ROS and a dramatic decrease in apoptosis of BV-2 microglia, which indicate that curcumin may offer neuroprotective effects by inhibiting oxidative damage to microglia (Yue et al., 2014). Curcumin was also reported to protect against the loss of retinal ganglion cells in the same chronic high intraocular pressure model (Yue et al., 2014). In another research, staurosporine (SS)-induced ganglion cell death was attenuated by low dosages of curcumin (<50 M) both *in vitro* and *in vivo* (Burugula et al., 2011). Acute retinal ischemia induced by high intraocular pressure followed by reperfusion (I/R) is an animal model for open-angle glaucoma. Dietary curcumin was reported to have a neuroprotective effect for retinal I/R injury. One important pathogenic cause for glaucoma is mitochondrial dysfunction. Mitofusin 2 (mfn2), a mitochondrial fusion protein, is decreased after retinal I/R injury. Nuclear factor erythroid 2-related factor 2 (Nrf2) has a protective effect against oxidative stress, and is increased after the retinal I/R injury. Pretreat of curcumin may reverse the decrease of mfn2 and the increase of nuclear factor erythroid 2-related factor 2 (Nrf2) in the retinal I/R-induced open-angle glaucoma model *in vivo*, indicating that curcumin could maintain the normal mitochondrial function and alleviate the retinal I/R injury by regulating the antioxidant system (Wang et al., 2011). Another important pathogenic cause for glaucoma is excitotoxicity (Wax and Tezel, 2002). Pretreat of curcumin significantly attenuates *N*-methyl-D-aspartate (NMDA)-induced apoptosis in retinal neuronal/glial cultures *in vitro* by inhibiting the NR1 subunit of the NMDA receptor (NMDAR) phosphorylation and NMDAR-mediated Ca^2+^ increase, demonstrating that curcumin possesses neuroprotective effects (Matteucci et al., 2005). Thus, curcumin could be a potential treatment strategy for glaucoma.

## Conclusions and Outlook

**FIGURE 2:**
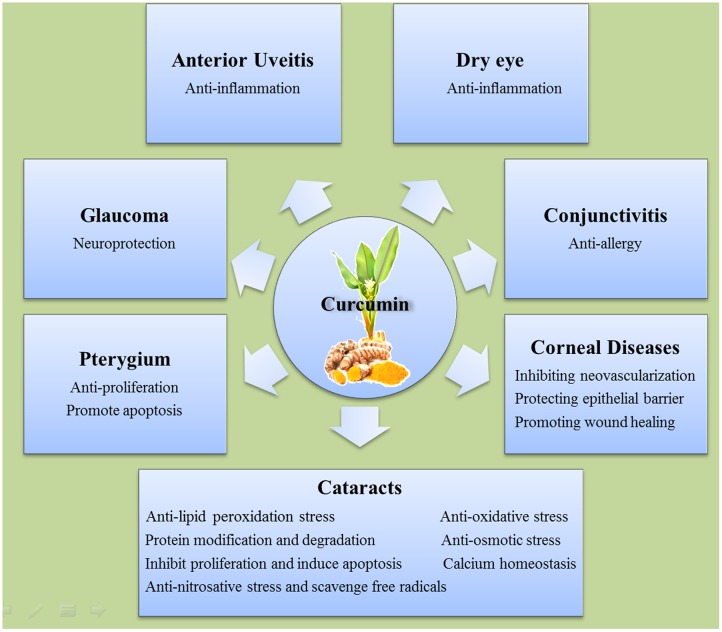
**The mechanisms of the beneficial effects of curcumin on different anterior segment eye diseases**.

Since ancient time, curcumin has been used in cooking and traditional medicine in both China and India. Modern science has illuminated many molecular basics for which the pharmaceutical use of curcumin may aid with human ailments. The selected observational studies on the relationship between curcumin and anterior segment eye diseases were summarized in **Table 2**. The mechanisms underlying the beneficial effects of curcumin on anterior segment eye diseases are summarized in **Figure 2**. *In vitro, in vivo*, and human clinical studies have suggested that curcumin has a diverse range of molecular targets, supporting the concept that it interacts with multiple cellular signaling pathways and modulates numerous molecular targets. Curcumin’s harmless nature, low cost, and multiple targeting potential make it a promising agent for the prevention and treatment of various eye diseases. Accumulating evidence has demonstrated its potential therapeutic value. Nevertheless, more randomized clinical trials are needed in order to solidify our understanding of its therapeutic potential.

**Table 2:** Selected observational studies on the relationship between curcumin and anterior segment eye diseases.

Region	Models	Oberservation	Results	Reference
Cornea	Cultured HCE	Effect of curcumin on TNF-α induced corneal barrier disruption	Curcumin blocked the TNF-α induced occludens-1 disappearance by suppressing the NF-κB pathway, and it also blocked TNF-α decreased TER.	Kimura et al., 2008
	Cultured HCE	Effect of curcumin on IL-1β induced corneal barrier disruption	Curcumin blocked the effects of IL-1β on occludens-1 and occludin by suppressing the NF-κB pathway, and it also blocked IL-1β decreased TER.	Kimura et al., 2009
	Cultured HCE	Effect of curcumin on dry eye disease	Curcumin inhibited hyperosmoticity-induced IL-1β elevation in HCE through P38 MAPK/NF-κB pathways.	Chen et al., 2010
	Mouse model of corneal NV, cultured bovine capillary endothelial cells, MS1 endothelial cells line	Effect of curcumin on the proliferation of endothelial cells with bFGF, and bFGF-induced corneal NV.	Curcumin inhibited bFGF-induced corneal NV in the mouse cornea and both endothelial cells’ proliferation.	Arbiser et al., 1998
	Rabbit and mouse model of corneal NV	Effects of curcuminoids on FGF-2-induced corneal NV	Localized and systemic delivery of curcuminoids inhibited the angioproliferative response to FGF-2 stimulation in rabbit and mouse corneas.	Mohan et al., 2000
	Rabbit model of corneal NV	Effect of curcumin on suturing-induced corneal NV	Topically curcumin inhibited suturing-induced corneal NV and VEGF mRNA upregulation.	Kim et al., 2010
	Aortic ring assay, rat model of corneal NV	Effect of curcumin NPs on silver nitrate-induced corneal NV	Topically curcumin NPs suppressed the expression of VEGF, inflammatory cytokines, and MMP. It prevented corneal NV by suppressing the NF-κB pathway.	Pradhan et al., 2015
	STZ-induced diabetic mice model with corneal epithelium abrasion	Effect of nanomicelle curcumin on corneal epithelial wound healing	Intranasal nanomicelle curcumin effectively promoted corneal epithelial/nerve wound healing in diabetic mice.	Guo et al., 2016
Conjunctiva	Patients	Effect of Curcuma-Longa on bacterial conjunctivitis	Curcuma-Longa had an active role on *E. Coli*,*St. Aureus*, Klebshella and pseudomonas organisms.	Srinivas and Prabhakaran, 1989
	Mice model of AC	Effect of curcumin on ovalbumin-induced AC	Curcumin inhibited the ovalbumin-induced iNOS activation, IL-4 and IL-5 production in the mice conjunctiva.	Chung et al., 2012
Conjunctiva/Cornea	Cultured human pterygium fibroblasts	Effect of curcumin on pterygium fibroblasts	Curcumin stopped the proliferation and caused the cell death of human pterygium fibroblasts.	Zhang et al., 2007
Uvea	Patients	Effect of curcumin on chronic anterior uveitis	Orally curcumin improved patients’ chronic anterior uveitis with improved vision, decreased aqueous flare, and keratic precipitates.	Lal et al., 1999
	Patients	Effect of curcumin-phosphatidylcholine complex on recurrent anterior uveitis	Orally curcumin-phosphatidylcholine complex improved recurrent anterior uveitis in more than 80% of patients.	Allegri et al., 2010
	*E. coli* lipopolysaccharide-induced rat uveitis	Effect of Curcuma-longa on endotoxin-induced uveal inflammation	Topical Curcuma-longa suppressed *E. coli* lipopolysaccharide-induced uveitis in rats by reducing TNF-α activity.	Agarwal et al., 2013
	*E. coli* lipopolysaccharide-induced rat uveitis	Effect of topical Curcuma-longa on endotoxin-induced uveal inflammation	Topical Curcuma-longa showed anti-inflammatory activity against endotoxin-induced uveitis in rabbits.	Gupta et al., 2008
Lens	see **Table 1**			
Neuronal/Glial	NMDA treated cultured retinal neuronal/glial cells	Effect of curcumin on retinal neuronal/glial cultures	Curcumin attenuates NMDA-induced apoptosis in retinal neuronal/glial cultures by inhibiting the phosphorylation of the NR1 subunit of the NMDA receptor, showing curcumin possed neuroprotective effects by inhibiting NMDA mediated excitotoxicity.	Matteucci et al., 2005
	Cultured BV-2 microglia cell line, rat model of chronic high intraocular pressure	Neuroprotective effect of curcumin on H_2_O_2_ treated BV-2 microglia cell line and microglia under chronic high intraocular pressure	Curcumin increased the cell viability of H_2_O_2_-treated BV-2 microglia and decreased the intracellular ROS and apoptosis. It protected microglia from death in chronic high intraocular pressure rat model. In both models, caspase 3, cytochrome c, and BAX were downregulated and BCL2 was upregulated in the curcumin-treated group.	Yue et al., 2014
	Staurosporine treated transformed mouse RGC-5 and mice	Effect of curcumin on death of retinal ganglion cells	Curcumin attenuated RGC and amacrine cell loss, by restoring NF-κB expression.	Burugula et al., 2011
	Rat model of acute retinal I/R injury	Effect of curcumin on retinal I/R injury	Curcumin could reverse the decrease of mfn2 and the increase of Nrf2 in the retinal I/R-induced glaucoma model. It protected retinal neurons and microvessels against I/R injury, may occur through its inhibitory effects on injury-induced activation of NF-κB and STAT3, and over-expression of MCP-1.	Wang et al., 2011


*NV, neovascularization; HCE, human corneal epithelial cells; TER, transepithelial electrical resistance; RGC-5, retinal ganglion-like cells; Streptozotocin, STZ; AC, allergic conjunctivitis.*

## Author Contributions

The topic was conceptualized by CL. CL, XL, DZ, and TX contributed to the literature database search, and writing of the manuscript. JH and NM contributed to vital revising. WZ and TM contribute to English Polishing.

## Conflict of Interest Statement

The authors declare that the research was conducted in the absence of any commercial or financial relationships that could be construed as a potential conflict of interest.

## Abbreviations

AR: Aldose reductase
CNLC: cationic nanostructured lipid carriers
GSH: Glutathione
GST: Glutathione *S*-transferase
IL-1β: interleukin-1 beta
I/R: ischemia/reperfusion
LPO: Lipoperoxides
MMP: matrix metalloprotein
NF-κB: nuclear factor kappa-light-chain-enhancer of activated B cells
NPs: nanoparticles
NSAIDs: Non-Steroidal Anti-inflammatory Drugs
NV: neovascularization
STZ: streptozotocin
TNF-α: tumor necrosis factor-α
VEGF: vascular endothelial growth factor
